# One Thousand Dollars Reward

**Published:** 1877-05

**Authors:** 


### One Thousand Dollars Reward.

With the view that Physicians may have examined for insanity, or if dead, may have made examination for cause of death, the following offer of *Reward* is published in our JOURNAL by request of Mrs. Dunlap and her friends.

*To Physicians and others in attendance at Hospitals, Asylums, and County Houses:*

**$1000 REWARD.**

I, MARIA DUNLAP, wife of Robert Dunlap, at Lockport, N. Y., will pay *One Thousand Dollars Reward* to any person who will bring said Robert Dunlap alive to his home, at Lockport. *Three Hundred Dollars Reward* to any person who will give to me information of his whereabouts, that may lead to his recovery and return; or who shall discover and return his body, if dead.

Dated Lockport, N. Y., Dec. 1, 1876. MARIA DUNLAP.

REFERENCE.

Hon. S. R. DANIELS, Mayor, Lockport, N. Y.
Hon. JAMES JACKSON, Jr., President Farmers and Mechanics Savings Bank, Lockport, N. Y.
JOHN T. MURRAY, U. S. Com'r, and Attorney-at-Law, Lockport, N. Y.
E. C. WRIGHT, Dealer in Books, Stationery, etc., Lockport, N. Y.
E. W. WILLIAMS, Supt. Canals State New York, Lockport, N. Y.
JOHN HODGE, Sec'y Merchant's Gargling Oil Co., and Pres't Union Printing and Pub. Co., Lockport, N. Y.

The following is a description of his person:

"Hon. Robert Dunlap, Lockport, N. Y., aged 60 years, 6 feet or over, about 200 pounds, black hair, mixed with grey, full beard, (except a mustache,) cut short, florid, weather-bronzed complexion, wearing a black slouch felt hat, faded blue overcoat, dark or black pants, dark or mixed cloth undercoat, and blue vest, all of well worn business clothing."

He left his home on Wednesday, Nov. 15th, 1876, at 8 A. M. When he left he was, beyond a doubt, partially insane, or acting under the delusion that the condition of certain business and pecuniary matters in which he was officially interested, was such that concealment or flight was necessary to his safety. There was no cause whatever for such belief.

